# Supplementary material for: Identification of photoperiod-induced specific miRNAs in the adrenal glands of Sunite sheep (Ovis aries)
Source: Front Vet Sci. 2022 Jul 22;9:888207. doi: 10.3389/fvets.2022.888207 (PMC9354845; doi:10.3389/fvets.2022.888207)
Supplement: Supplementary file 1 [file Data_Sheet_1.ZIP › Supplementary file/miRDeep2 file.zip!/miRDeep2 file/Know_Structure/SPLP42A1/oar-mir-410.pdf]

miRBase precursor : oar-mir-410
Total read count : 960
oar-miR-410-5p read count : 0
oar-miR-410-3p read count : 956
remaining reads : 4

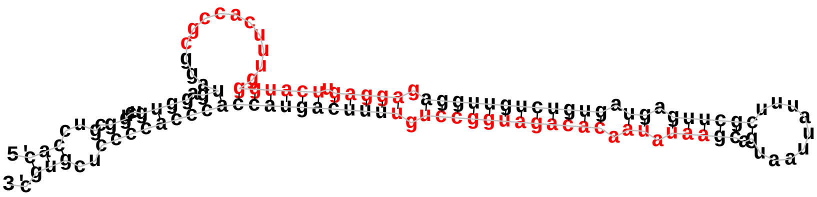

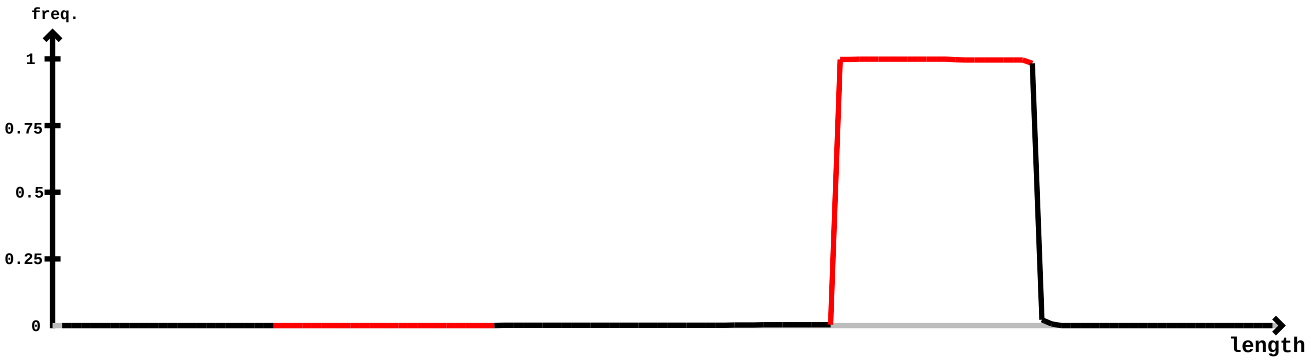


```
                                    oar-miR-410-5p
                                                                                 oar-miR-410-3p
5'- caccugcggucuguggaguaggcgccacuuuggguacuugaggagagguugucugugaugaguucgcuuuauuaaugacgaauauaacacagauggccuguuuucaguaccacccaccccucgugc  -3'   exp
    (((..(.(((..((((........))))....((((((.(((((.(((((((((((((.((.((((((.........).))))).)).))))))))))))).)))))))))))....))).)..))).  reads   mm
    .............................................agguugucugugaugaguucg............................................................  1       0
    ..................................................................cuuuauuaaugacgaauauaacacaga.................................  1       0
    .....................................................................uauuaaugacgaauauaacacag..................................  1       0
    ........................................................................uaaugacgaauauaacacag..................................  1       0
    ................................................................................aauauaacacagauggccug..........................  12      0
    ................................................................................aauauaacacagauggccugu.........................  923     0
    ................................................................................aauauaacacagauggccuguu........................  15      0
    ................................................................................aauauaacacagauggccuguuu.......................  5       0
    ..................................................................................uauaacacagauggccuguuu.......................  1       0
```
